# Costs of Dengue Control Activities and Hospitalizations in the Public Health Sector during an Epidemic Year in Urban Sri Lanka

**DOI:** 10.1371/journal.pntd.0004466

**Published:** 2016-02-24

**Authors:** Neil Thalagala, Hasitha Tissera, Paba Palihawadana, Ananda Amarasinghe, Anuradha Ambagahawita, Annelies Wilder-Smith, Donald S. Shepard, Yeşim Tozan

**Affiliations:** 1 National Child Health Programme, Family Health Bureau, Ministry of Health, Colombo, Sri Lanka; 2 Epidemiology Unit, Ministry of Health, Colombo, Sri Lanka; 3 National Dengue Control Unit, Colombo, Sri Lanka; 4 Department of Public Health and Clinical Medicine, Epidemiology and Global Health, Umeå University, Umeå, Sweden; 5 Lee Kong Chian School of Medicine, Nanyang Technological University, Singapore, Singapore; 6 Brandeis University, The Heller School for Social Policy and Management, Waltham, Massachusetts, United States of America; 7 Institute of Public Health, Heidelberg University Medical School, Heidelberg, Germany; 8 College of Global Public Health, New York University, New York, New York, United States of America; Florida Department of Health, UNITED STATES

## Abstract

**Background:**

Reported as a public health problem since the 1960s in Sri Lanka, dengue has become a high priority disease for public health authorities. The Ministry of Health is responsible for controlling dengue and other disease outbreaks and associated health care. The involvement of large numbers of public health staff in dengue control activities year-round and the provision of free medical care to dengue patients at secondary care hospitals place a formidable financial burden on the public health sector.

**Methods:**

We estimated the public sector costs of dengue control activities and the direct costs of hospitalizations in Colombo, the most heavily urbanized district in Sri Lanka, during the epidemic year of 2012 from the Ministry of Health’s perspective. The financial costs borne by public health agencies and hospitals are collected using cost extraction tools designed specifically for the study and analysed retrospectively using a combination of activity-based and gross costing approaches.

**Results:**

The total cost of dengue control and reported hospitalizations was estimated at US$3.45 million (US$1.50 per capita) in Colombo district in 2012. Personnel costs accounted for the largest shares of the total costs of dengue control activities (79%) and hospitalizations (46%). The results indicated a per capita cost of US$0.42 for dengue control activities. The average costs per hospitalization ranged between US$216–609 for pediatric cases and between US$196–866 for adult cases according to disease severity and treatment setting.

**Conclusions:**

This analysis is a first attempt to assess the economic burden of dengue response in the public health sector in Sri Lanka. Country-specific evidence is needed for setting public health priorities and deciding about the deployment of existing or new technologies. Our results suggest that dengue poses a major economic burden on the public health sector in Sri Lanka.

## Author Summary

Dengue is a major public health problem affecting more than half of the world’s population living in tropical and subtropical regions of the world. The disease is estimated to place a heavy socio-economic burden on households, health care systems, and governments, particularly during outbreaks; however, country-specific reliable estimates of burden of disease and cost data are limited. This study estimated the public sector costs of dengue control activities and the direct costs of hospitalizations in Colombo district—the most heavily populated and urbanized district in Sri Lanka—during the epidemic year of 2012 from the Ministry of Health’s perspective. Results revealed that the Ministry’s cost of dengue control and hospitalizations totaled US$3.45 million (US$1.50 per capita), of which US$971,360 (US$0.42 per capita) was for dengue control activities. Personnel costs accounted for the largest shares of the costs of dengue control activities (79%) and of hospitalizations (46%). The results indicated a per capita cost of US$0.42 for dengue control activities. The average costs of hospitalization ranged between US$216–609 for pediatric cases and between US$196–866 for adult cases according to disease severity and treatment setting. These results suggest that dengue poses a major economic burden on the public health sector in Sri Lanka.

## Introduction

Dengue is a major public health problem affecting more than half of the world’s population living in tropical and subtropical regions of the world [1], particularly in Asia, which bears about 70% of the estimated 390 million apparent dengue infections annually [2]. A good proportion of these infections, especially in children, progress from mild dengue fever (DF) to more severe and life-threatening disease known as dengue hemorrhagic fever (DHF). Commensurate with increases in the size, duration and frequency of dengue epidemics, the incidence of DF and DHF and the rate of dengue hospitalizations have increased markedly in the past few decades [3–5], most noticeably in Southeast Asia [6]. Given the multitude of public health priorities, dengue represents a formidable development challenge to affected countries with developing economies; there are limited public health resources and infrastructure to contain dengue transmission and spread in rapidly urbanizing settings under population pressure. The disease is estimated to place heavy socio-economic burden on households, health care systems, and governments [7–9], particularly during outbreaks [10]; however, country-specific reliable estimates of burden of disease and cost data are limited [1, 11].

Reported as a public health problem since the 1960s [12], dengue is considered to be hyperendemic in Sri Lanka with all four distinct serotypes of the dengue virus in circulation for more than 30 years [13]. Historically, dengue was a less serious public health threat because of low incidence of severe disease with regular but small epidemics, restricted to the highly populated and urbanized southwestern region of the island [12, 14–16]. An abrupt increase in the number of DHF cases occurred in 1989 [12] and then again in 2000 [14], owing to the emergence of new genotypes of the dengue virus and the geographic expansion of dengue vectors [13, 17]. Dengue became a notifiable disease in 1996 [18], and a National Dengue Control Program was set up at provincial and district levels in 1998 [15]. Nonetheless, the number of reported cases of DF and DHF has increased 10-fold since the early 2000s [13]. Large epidemics are now occurring at regular intervals, affecting the entire population of the island [19]. Dengue has become a high priority disease for public health authorities in Sri Lanka.

The Sri Lankan Ministry of Health is responsible for controlling dengue and other disease outbreaks and associated health care. The main pillar of dengue prevention is currently limited to the control of mosquito vectors [20]. The involvement of large numbers of public health staff in year-around dengue control activities and the provision of free medical care to dengue patients at public hospitals place a formidable financial burden on the Ministry of Health. The aim of this study was to estimate the public sector costs of dengue control activities and the direct costs of hospitalizations in Colombo district—the most heavily populated and urbanized district in Sri Lanka—during the epidemic year of 2012. The costing was conducted from the Ministry of Health’s perspective and focused on operational and administration costs. The findings are expected to inform public health planning and decision-making in Sri Lanka and contribute to the growing body of literature on the costs of dengue prevention and treatment [1], particularly in Southeast Asia in view of longer and more frequent cyclical epidemics in the region [6].

## Methods

### Study setting

**Fig 1:**
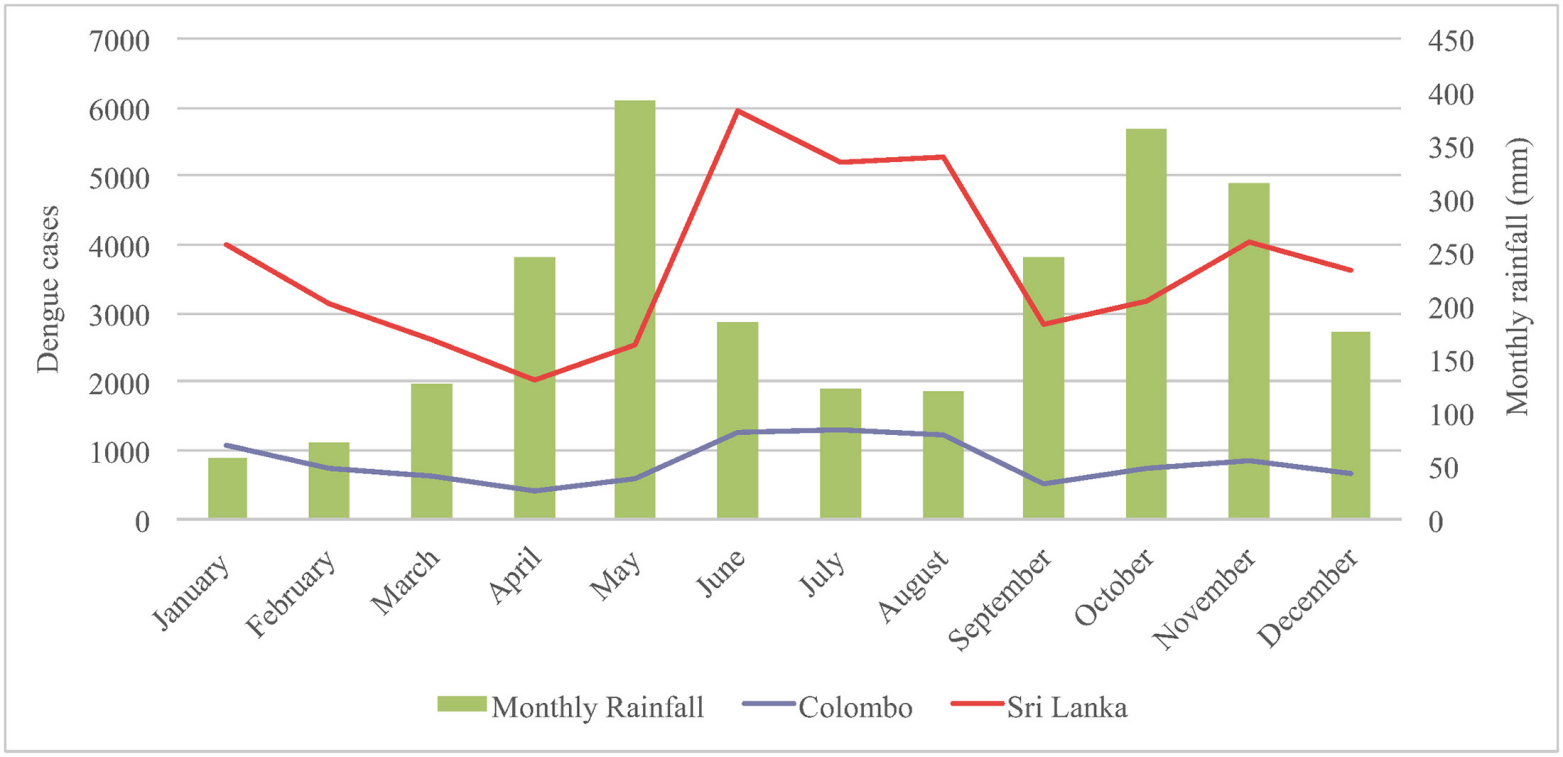
Number of reported dengue cases and average monthly rainfall in Colombo district, Sri Lanka, 2012.

We conducted this costing study in Colombo district, which is located in the most heavily urbanized Western province in Sri Lanka. Colombo has a population of 2.3 million, accounting for 11.5% of the total population of the country [21]. Colombo and its neighboring districts are located in the wet zone of the island and report the highest numbers of dengue cases every year [22]. The number of reported cases usually peaks in June during the southwesterly monsoon (May-August). Dengue is, however, an all-year-around disease in Sri Lanka. During the epidemic year of 2012, 10,017 hospitalized dengue cases were reported from secondary care public hospitals in Colombo, corresponding to an incidence rate of 435 per 100,000 population for the district and accounting for 22.5% of the total reported cases in the country (Fig 1) [23].

### Public health sector involvement in dengue control and associated health care

**Fig 2:**
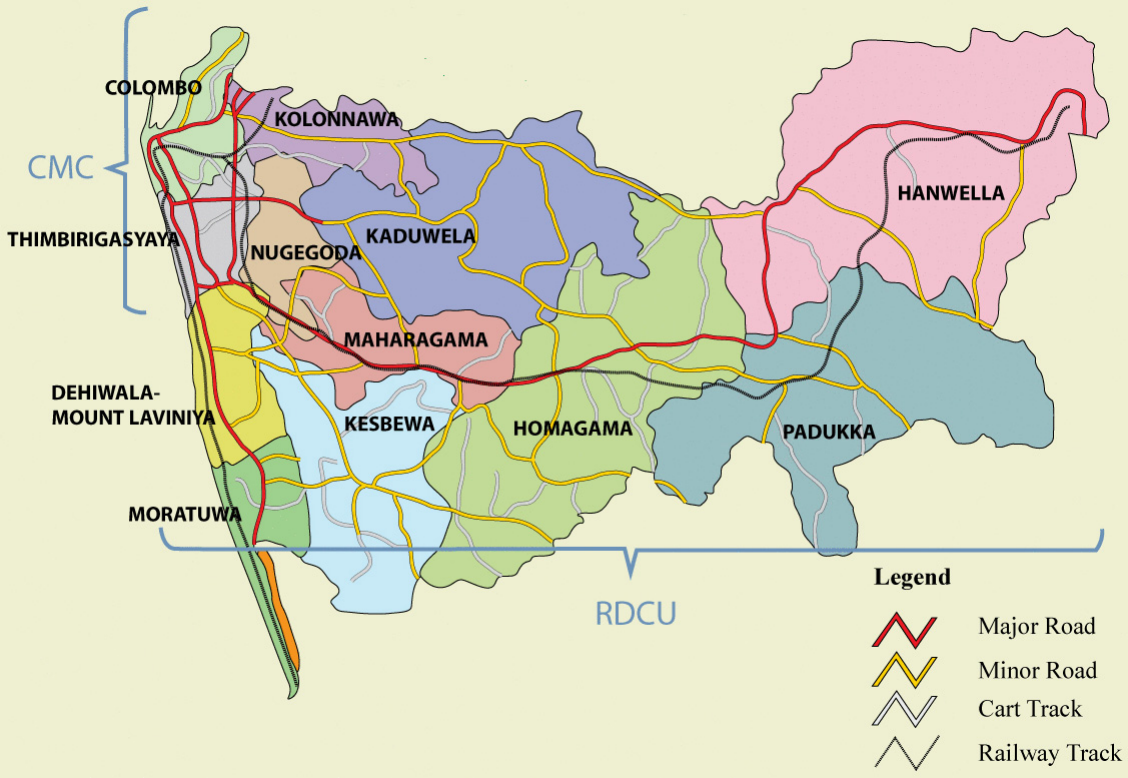
Map of Medical Officer of Health and Divisional Secretary areas in Colombo district, Sri Lanka, with public health agencies responsible for dengue control activities.

The basic health care unit at the sub-district level is a Medical Officer of Health (MOH) area. Colombo district is sub-divided into 12 MOH areas across 13 Divisional Secretary (DS) areas (Table 1). Dengue control activities operate under the administrative purview of the Regional Dengue Control Unit (RDCU) attached to the Regional Directorate of Health (RDH) in all DS areas, except in Colombo and Thimbirigasyaya where a separate dengue control unit in the Health Department of the Colombo Municipal Council (CMC) is the public health agency responsible for the activities (Fig 2). The RDCU supports, coordinates, and monitors and evaluates all dengue control activities in the MOH areas under its purview with a team fully dedicated to dengue control, comprising 2 Medical Officers, 1 Entomologist, 1 Epidemiologist, 1 Public Health Inspector (PHI), 2 Public Health Field Officers (PHFOs), 3 Spray Machine Operators (SMOs), 100 Health Assistants, 1 Management Assistant and other ancillary staff (2 drivers and 2 laborers). The RDCU also provides technical training to MOH staff and enforces the mosquito control legislation in MOH areas through the use of fines.

**Table 1:** Public health sector agencies in charge of dengue control activities in Colombo District, Sri Lanka.

Public health sector agency	DS areas	MOH areas
RDH Colombo—RDCU	Dehiwala—Mt. Lavinia	Dehiwala—Mt. Lavinia
	Moratuwa	Moratuwa
	Kolonnawa	Kolonnawa
	Nugegoda	Kotte
	Maharagama	Maharagama
	Kesbewa	Piliyandala
		Boralesgamuwa
	Kaduwela	Kaduwela
	Homagama	Homagama
	Padukka	Padukka
	Hanwella	Hanwella
CMC Health Department	Colombo	CMC
	Thimbirigasyaya	

DS = Divisional Secretary; MOH = Medical Officer of Health; RDH = Regional Directorate of Health; RDCU = Regional Dengue Control Unit; CMC = Colombo Municipal Council

In MOH areas, a team of trained public health staff undertakes a set of primary health care services, including dengue prevention, under the supervision of an MOH (i.e. a community physician). Across all MOH areas, a total of 35 MOHs, 12 Supervising Public Health Inspectors (SPHIs), 82 PHIs, 20 SMOs, 12 drivers and 12 laborers are involved with dengue control activities. MOH teams are assisted by local government employees, police forces, school children and public volunteers in the field. Community-level dengue activities include periodic inspection of households and public places to identify and remove potential mosquito breeding places, vector control activities such as fogging with insecticides and biological and chemical larviciding, and public awareness campaigns where residents are encouraged to clean breeding sites and are made aware of the mosquito control legislation and its implications. Litter cleanup campaigns are also conducted routinely along with larviciding with the participation of local government workers under the supervision of PHIs.

The national dengue response includes systematic vector and disease surveillance activities. Vector surveillance is conducted by the Entomological Unit of the RDCU, which regularly inspect breeding sites, conduct larval surveys at sentinel sites, and inform vector control activities in MOH areas accordingly. The National Communicable Disease Surveillance System in Sri Lanka legally mandates secondary care public hospitals to notify all dengue cases seeking inpatient care to relevant MOH office based on the residence of patients and to the central Epidemiology Unit attached to the Ministry of Health. Dengue notification initiates a series of actions in MOH areas in which PHIs and MOHs visit the households to confirm cases and identify potential sources of infection. PHIs also survey surrounding households to gauge the risk of dengue in the area and organize vector control activities as necessary. MOHs compile and submit weekly reports on dengue and other notifiable diseases (number of cases and deaths) to the Epidemiology Unit.

The Colombo district has 57 primary and 7 secondary care public hospitals. Only secondary care public hospitals provide inpatient care in the district, and these hospitals collectively have 1,615 adult and 711 pediatric inpatient beds. One of them is a pediatric hospital with 474 beds. Almost all dengue patients in need of hospitalization access treatment at a secondary care public hospital. Ambulatory dengue patients can seek care at public hospitals as well as private general practitioners and hospitals. At present the official reporting system run by the Ministry of Health captures relatively few ambulatory dengue cases. Therefore, this cost analysis was limited to hospitalized dengue cases.

### Cost of dengue control activities

The financial costs borne by the public health agencies in charge of dengue control activities in Colombo district during the epidemic year of 2012 were collected and analyzed retrospectively. Costs external to the public health sector (e.g. non-health public sectors, private sector, and community) were not included in the analysis. The basic task of any costing analysis is to identify, measure and value activities. Therefore we first conducted interviews with public health staff involved with dengue control at the RDCU and MOH level and reviewed available documentation to identify the activities (Table 2). We then developed a cost extraction tool to capture the costs of these activities. SPHIs in each MOH area were trained to use the cost extraction tool and collected cost data directly from the financial records of MOH offices. The accuracy of the extracted cost data was validated using the financial records kept at the RDCU, which received routine activity and expenditure reports from MOH offices. The costs of dengue control activities in the CMC MOH area were extracted from the annual compilation sheets of the Health Department of the CMC. We did not have access to detailed resource use information or cost data because dengue control in the CMC area does not fall under the purview of the RDH. This limited the feasibility of data collection.

**Table 2:** Cost composition of dengue control activities in Colombo District, Sri Lanka, 2012 (all costs are in US$).

	Total	%
Recurrent costs		
Personnel	770,518	79
Consumables (larvicides, insecticides, kerosene oil, chemicals, petrol, diesel)	150,871	16
Utilities (electricity, water, communication) and maintenance (equipment and machinery, vehicles, infrastructure facilities)	27,270	3
Sub-total recurrent	948,659	98
Capital costs (amortized)		
Equipment and machinery (fogging machines, spraying machines, personal protective gear, laboratory equipment)	22,701	2
Sob-total capital	22,701	2
Overall cost	971,360	100

We used a combination of activity-based costing and ingredients approach in which all resources used for the planning and implementation of dengue control activities were identified, measured, and valued [24]. Costs were divided into recurrent and capital costs [25]. The recurrent costs included personnel (salaries and allowances), consumables (larvicides, insecticides, kerosene oil, chemicals, petrol, and diesel), maintenance of equipment and machinery, vehicles, infrastructure facilities and utilities (water, electricity, communication). Except for SMOs, MOH staff was involved with the provision of a variety of primary health care services. 12 MOHs, 12 SPHIs and 24 PHIs determined the proportion of time they spent on dengue control activities over a period of one month using a work diary developed specifically for this purpose. This information was extrapolated to one year [26]. The MOH personnel costs were apportioned according to measured staff time spent on dengue control activities against all other primary health care activities. The RDCU staff worked exclusively on dengue. Therefore no adjustment was made to the RDCU personnel costs. MOH staff trainings were carried out by RDCU staff, and associated time costs were included in MOH and RDCU personnel costs. No additional expenditures were reported for training activities during the study period.

The costs of consumables were calculated by multiplying the quantities used during the study period by their unit costs, valued at local market prices. Maintenance (equipment and machinery, vehicles, infrastructure facilities) and utility costs (water, electricity, communication) were available on an annual basis for each MOH office. These costs (except equipment and machinery used exclusively for dengue control) were apportioned according to the MOH time spent on dengue control activities. The maintenance and utility costs of the RDCU office was fully included.

The capital costs included fogging machines, spraying machines, personal protective gear and laboratory equipment such as microscopes and pipettes. Buildings and vehicles were not included in the capitals costs because of lack of data on their replacement costs. The annualized values of the capital items were estimated using a standard procedure, assuming an appropriate useful life year for each item and using a discount rate of 3% [27].

### Cost of dengue hospitalizations

Of the seven secondary care public hospitals in the Colombo district, three participated in the costing analysis of the medical costs of dengue patients. A random sample of 100 adult and pediatric patients who received inpatient care for dengue in 2012 was selected from the patient records of participating hospitals. All hospitalized dengue cases were clinically diagnosed. A team of pre-intern medical doctors was trained to extract data from the bed-head tickets of patients using a data extraction form designed specifically for the study. The extracted data for each patient included the final clinical diagnosis of dengue (DF or DHF), the quantity and type of drugs (oral and parenteral) and medical supplies used, the number and type of laboratory investigations performed, the type of hospital ward (dengue ward and intensive care unit, ICU) to which the patient was admitted, and the length of hospital stay. Using this data, an average medical cost per case (DF or DHF) was calculated by treatment setting (dengue ward or ICU) for pediatric and adult patients. Medical cost estimates did not include the costs of hospital staff, which were estimated separately as part of the cost of hospital stay at a secondary care public hospital. The source of unit costs for all drugs and medical supplies was the MoH Drugs and Supplies Unit. Private sector costs of laboratory investigations were used in the absence of relevant cost data from the participating hospitals.

Additional cost data were collected retrospectively from the operating budget of all seven secondary care hospitals in Colombo district and analyzed using a gross-costing approach. We extracted hospital personnel costs (salaries, allowances and incentives for doctors, nurses and other hospital staff), utilities (communication, electricity, water), operational costs (laundry, security, meals), and maintenance costs (repair and maintenance of equipment and buildings). These costs were adjusted according to the total dengue patient days at each hospital during the study year and summed up across seven hospitals. Assuming all hospitalized dengue patients were average in respect to these three cost categories, a fixed cost of hospital stay per dengue patient was calculated on the basis of total number of dengue patients received inpatient care at these hospitals during the study year. To estimate the average costs of hospitalized DF and DHF cases, the fixed cost of hospital stay was then combined with the average medical cost per case according to treatment setting. The average costs of dengue hospitalization were estimated for adult and pediatric patients separately.

In the Colombo district, a total of 10,017 dengue cases were notified from seven secondary care public hospitals in 2012. We divided the total notified cases into pediatric and adult patients, and then subdivided them into DF and DHF cases based on the surveillance data from three sentinel hospitals in the district in 2012. Sentinel hospital data showed that 1% of adult and 2% of pediatric dengue patients were treated in an ICU [28]. The total direct cost of dengue hospitalizations in the Colombo district during the epidemic year of 2012 was then estimated by multiplying the average cost of a hospitalized dengue case by the estimated number of cases (DF or DHF) in pediatric and adult patients.

All costs were identified in local currency, Sri Lankan Rupees (LKR), and later converted to United States Dollars (US$) according to the average exchange rate for 2012, 1 USD = 131 LKR. Microsoft Excel was used for the cost analysis.

As we studied only a single year (2012), there was no variation in the costs of dengue control activities nor in the number of notified dengue cases. Because the major costs of hospitalization (personnel and infrastructure) were calculated in aggregate, we had to rely on supplemental data to estimate uncertainty in the cost per hospitalized case. We obtained the coefficient of variation in length of stay and cost per day in public hospitals from a study of hospitalized dengue cases in the neighboring country (India) in the same year with similar methods and overlapping authorship [29]. We assumed that the length of stay and cost per day were normally distributed and statistically independent and derived the uncertainty in cost per day based our total sample size of hospitalized cases.

### Ethics

This study received ethical approval from the institutional review board of the Sri Lankan Ministry of Health, and all data analyzed were anonymized.

## Results

The total public sector cost of dengue control activities in Colombo district during the epidemic year of 2012 was estimated at US$971,360. The cost composition of the activities is presented in Table 2. About 79% of these costs were personnel costs. This was followed by consumables costs, corresponding to 16% of total costs. These results indicated a per capita cost of US$0.42 for dengue control activities in Colombo district.

Table 3 shows the estimated medical costs for DF and DHF cases by treatment setting (dengue ward and ICU) for pediatric and adult patients in secondary care hospitals in Colombo district. The results show a clear escalation in the medical costs by disease severity and treatment setting. The medical costs also varied by patient category (pediatric and adult). Hospital personnel, utility, operational and maintenance costs amounted to a total operating cost of US$1,642,222, on the basis of 10,017 patients who received inpatient care at secondary care public hospitals in 2012. Assuming all hospitalized dengue patients were average in respect to these costs, an average public cost of hospital stay per dengue patient was estimated at US$164. Using this estimate, the average cost of hospitalization per DF and DHF case by treatment setting were derived for pediatric and adult patients (Table 4). Combining these data with the distribution of notified dengue cases by final diagnosis and treatment setting, we derived the total direct costs of dengue hospitalizations at secondary care hospitals in Colombo district in 2012, which amounted to US$2.5 million (Table 5). The cost composition of dengue hospitalizations in the public sector is presented in Table 6. The key cost drivers were hospital personnel costs (US$1,131,661; 46%) and medical costs (US$840,258; 34%), followed by hospital utility and maintenance costs (US$510,561; 21%). Of the total medical costs, 71% were for clinical case management of DHF patients, amounting to US$599,187.

**Table 3:** Average medical costs ± standard error of the mean per pediatric and adult case at secondary care public hospitals in Colombo district, 2012 (all costs are in US$).

Patient category	Final diagnosis	Treatment setting	
		Dengue ward	ICU
Pediatric (≤12 years of age)	DF	51±1	79±2
	DHF	129±3	445±11
Adult (>12 years of age)	DF	32±1	330±8
	DHF	91±2	702±18

DF = Dengue fever; DHF = Dengue Hemorrhagic Fever; ICU = Intensive care unit

**Table 4:** Average cost of hospitalization ± standard error of the mean per pediatric and adult case at secondary care public hospitals in Colombo district, Sri Lanka, 2012 (all costs are in US$).

Type of cost	Pediatric (≤12 years of age)				Adult (>12 years of age)			
	DF		DHF		DF		DHF	
	Dengue Ward	ICU	Dengue Ward	ICU	Dengue Ward	ICU	Dengue Ward	ICU
Average medical cost	51±1	79±2	129±3	445±11	32±1	330±8	91±2	702±18
Average cost of hospital stay	164±4	164±4	164±4	164±4	164±4	164±4	164±4	164±4
Average cost of hospitalization	215±5	243±6	293±7	609±15	196±5	494±12	255±6	866±22

DF = Dengue fever; DHF = Dengue Hemorrhagic Fever; ICU = Intensive care unit

**Table 5:** Total cost of dengue hospitalizations ± standard error of the mean in Colombo district, Sri Lanka, 2012 (all costs are in US$).

	Pediatric (≤12 years of age)				Adult (>12 years of age)			
	DF		DHF		DF		DHF	
	Dengue ward	ICU	Dengue ward	ICU	Dengue ward	ICU	Dengue ward	ICU
Number of dengue cases	3,732±93	8±0.2	2,747±69	124±3	1,504±38	6±0.2	1,868±47	28±1
Cost of hospitalization per case	215±5	243±6	293±7	609±15	196±5	494±12	255±6	866±22
Total cost	802,169±20,054	1,944±49	804,871±20,122	75,516±4,776	294,784±1,888	2,964±7,370	476,340±11,909	24,248±501,00
Overall cost	2,482,836±62,071							

DF = Dengue fever; DHF = Dengue Hemorrhagic Fever; ICU = Intensive care unit

**Table 6:** Composition of dengue hospitalization costs ± standard error of the mean in the public health sector in Colombo district, Sri Lanka, 2012 (all costs are in US$).

Type of cost	Total	%
Medical costs	840,258±21,006	34
*Medical costs*: *DF/Pediatric*	*190*,*964±4*,*774*	*8*
*Medical costs*: *DHF/Pediatric*	*409*,*543±10*,*239*	*16*
*Medical costs*: *DF/Adult*	*50*,*108±1*,*258*	*2*
*Medical costs*: *DHF/Adult*	*189*,*644±4*.*741*	*8*
Hospital personnel costs (salaries and allowances)	1,131,661±28,292	46
Hospital utilities, operational and maintenance costs	510,561±12,764	21
Overall cost	2,482,836±62,071	100

DF = Dengue fever; DHF = Dengue Hemorrhagic Fever

The total cost of dengue control and hospitalizations borne by the public health agencies in Colombo district during the epidemic year of 2012 was estimated at US$3.45 million, corresponding to a per capita cost of dengue response of US$1.50 in the public health sector.

## Discussion

This analysis is a first attempt to assess the economic burden of dengue response in the public health sector in the heavily urbanized and densely populated Colombo district in Sri Lanka. We reported the actual use of financial resources by public health agencies for the provision of dengue control and hospital care services during the epidemic year of 2012. The total cost of dengue response of US$3.45 million is equivalent to the aggregate annual economic output of 1,182 people, based on a GDP per capita of US$2,922 in 2012. We found that personnel costs accounted for the largest share of the total costs of dengue control activities and hospitalizations—79% and 46%, respectively—highlighting the human resource intensive nature of these services, similar to other settings [30–34]. While Colombo is only one of the 25 districts affected by dengue in Sri Lanka, this study shows that the resources used for dengue prevention and hospital care services are substantial and represent an opportunity cost to the public health sector that can be reallocated to other health services.

The estimated US$0.42 per capita cost of dengue prevention and control in Colombo corresponds to a monthly cost of US$0.03 per capita, which falls below the range—US$0.13 per month in Panama [35] to 1.88 per month in Cuba [30]—reported by other studies during dengue outbreaks. This can be explained by some of the limitations of this costing study. First, costs for dengue prevention in non-health public sectors, private sector, and households and communities were not included because of the Ministry of Health perspective of the analysis. Second, the costing study focused on operational and administration costs and did not measure and value in-kind contributions such as materials, supplies, and volunteer time devoted to community-level dengue control activities. Third, some of the capital costs such as buildings and vehicles were omitted due to lack of information on their replacement costs. Nevertheless, there are considerable investment costs associated with setting up large-scale dengue control and treatment programs. Such investment costs are often difficult to capture and quantify in costing analyses, and can be substantially greater than initial capital outlay and vary significantly from one setting to another.

The average costs of dengue hospitalization at secondary care hospitals ranged between US$216–609 for pediatric cases and between US$196–866 for adult cases, according to disease severity and treatment setting. These estimates are comparable with the estimates from other studies [8, 36, 37]. For instance, the mean cost of hospitalization in the public sector was US$714 for patients under 15 years of age, US$272 for patients aged 15–60 years, and US$304 for patients over 60 years of age in Brazil [37]. Our study did not capture the costs of ambulatory care services in the public health sector, which was shown to be a major component of dengue treatment costs in other studies [8, 38, 39]. For instance, the average direct cost for ambulatory patients was about 20–25% of that of hospitalized patients in Mexico and Malaysia [8, 39, 40]. Applying this range to the average cost of hospitalization per DF case in a ward setting, the cost of ambulatory care services would be estimated to range between US$39.20–53.75 per dengue patient in our setting. A recent review estimated an average outpatient to inpatient (OP:IP) case ratio of 2.6 to allow for extrapolation from hospitalized episodes to total episodes, using available data from Southeast Asian countries [41]. Using this OP:IP case ratio, a total of 26,044 ambulatory dengue cases would be estimated with associated ambulatory care costs amounting to US$1,020,925–1,238,615. This corresponds to almost 50% of the estimated cost of dengue hospitalizations and suggests a potentially significant burden of ambulatory cases on the public health system.

Costs associated with dengue hospitalizations are incurred by not only public hospitals, but also households, employers and insurance companies [36, 39, 42]. These costs were not included in this analysis. While important for priority setting and planning and implementation purposes by public health authorities, our cost estimates are therefore conservative, understating the overall economic burden of dengue treatment in Colombo district during the study period.

In this study, we analyzed cost data only from the epidemic year of 2012. There can be great variation in dengue control program costs and health care expenditures from epidemic to non-epidemic years, owing to intensified prevention and control activities and increased hospitalization rates during epidemics. It is well documented that the number of dengue cases varies considerably from year to year. In Sri Lanka, the total number of reported dengue cases in the national surveillance system has increased substantially from 15,463 (80/100,000 population) in 2004 to 30,251 (142/100,000) in 2009 to 44,461 (218/100,000 population) in 2012 [23]. Disease notification is mandatory for public health facilities, but there may also be differences in reporting between epidemic and non-epidemic periods. Against this backdrop, more research summarizing the costs and the number of cases over a series of calendar years, with a specific focus on epidemic and non-epidemic years, is needed for a more comprehensive and dynamic picture of the economic burden of dengue in Sri Lanka.

Dengue is a rapidly expanding global public health problem with frequent cyclical epidemics. The increase in the incidence of DF and DHF will likely continue as we wait for the licensure of a safe and effective dengue vaccine to be deployed widely in endemic countries. Dengue prevention can result in a drastic reduction in the number of dengue patients seeking care in health facilities, reducing the pressure on over-stretched health systems and benefiting the entire health system. On the other hand, intensive vector control efforts can burden public health agencies. Global and country-specific evidence on the economic burden of dengue is needed for setting public health priorities and deciding about the deployment of existing or new technologies, but accurate estimation is fraught with challenges because of incomplete data. Our results and previous studies suggest that dengue poses a major economic burden to the public health sector, and more in-depth research is needed in Sri Lanka and other affected countries.
